# Saudi adults satisfaction with community pharmacy services

**DOI:** 10.1186/s40064-016-2442-8

**Published:** 2016-06-17

**Authors:** Mohamad Al-Tannir, Afnan I. Alharbi, Arwa S. Alfawaz, Razan I. Zahran, Mustafa AlTannir

**Affiliations:** Research Center, King Fahad Medical City, Riyadh, Saudi Arabia; College of Pharmacy, Princess Nora bint Abdulrahman University, Riyadh, Saudi Arabia; College of Medicine, AlFaisal University, Riyadh, Saudi Arabia

**Keywords:** Patient satisfaction, Community pharmacy services, Patient experience, Pharmacist–patient communication

## Abstract

**Background:**

Community pharmacists play a significant role in patient health care in Saudi Arabia and are directly responsible for medication-related counseling. Aim To assess Saudi Adults satisfaction with community pharmacy services and the secondary objective is obtaining an insight of their needs in patient counseling service.

**Methods:**

This cross-sectional survey was conducted via a questionnaire distributed to a representative sample of 650 Saudi adults approached at pharmacists and public places in areas of Riyadh during March 2014.

**Results:**

500 Complete questionnaires were collected, and the response rate was 77 %. Around 41 % were satisfied with Saudi pharmacy services. Out of these, 57 % attributed their satisfaction to pharmacist counseling on current medication, 96 % to appropriate dosage regimen explanation and 73 % to appropriate time spent in counseling (p < 0.001). When asked about reasons for dissatisfaction, 82 % of the unsatisfied group stated that pharmacists fail to ask about comorbid diseases and 78 % reported unavailability of dedicated pharmacist for patient counseling (p < 0.001).

**Conclusion:**

Saudi adults have variable levels of satisfaction with community pharmacy services. To increase levels of satisfaction, it is necessary to provide adequate pharmacist training in community pharmacies.

## Impact of findings on practice

- The patient-pharmacist relation has immense importance in improving patient health. This study highlights the areas that are lacking in pharmacy services and can be henceforth improved.
- Awareness about pharmacist responsibilities and patient satisfaction levels will lead to better pharmacy services and patient care.

## Background

Pharmaceutical care is the directly responsible provision of medication-related care to improve a patient’s quality of life (Slack and Ing 2009). The pharmacist has to establish a short-term relationship with the patient in order to ensure appropriate use of their medication.

The role of the pharmacist comprises of providing complete information and services that contribute to achieving the best anticipated results of the medications while minimizing, as much as possible, the occurrence of the untoward effects. Pharmacists’ role also encompasses increasing patients’ compliance with the medication, counseling on dosage regimen and providing instructions about the common side effects of both prescribed and over the counter medications (OTC) (Rayes et al. 2014; Gu et al. 2008). It is expected, from the point of view of the society, that pharmacists should have a definite and active role in patients’ satisfaction including counseling and providing comprehensive knowledge about medication use (Iqbal et al. 2008).

Patient satisfaction is a good indicator of healthcare quality (Mpinga and Chastonay 2011; Fenton 2012; Browne et al. 2010) which could be affected by patients’ adherence to their treatment regimen (Viktil et al. 2013). Patient satisfaction on the pharmacist’s performance can be affected by the pharmacist’s instructions about the use and adherence to medication (Kassam et al. 2012).

Patient satisfaction is of paramount importance for several reasons. High satisfaction levels indicate that community pharmacies are performing their role effectively, whereas, unsatisfied patients will be reluctant to visit the community pharmacy again if they were displeased in their previous experiences, leading to a loss of patients. On the other hand, poor satisfaction levels may act as a trigger that will motivate the pharmacists to improve the different aspects of patient services (Al-Arifi 2012).

Counseling is considered the most valuable service provided by community pharmacies. Some studies prove that the patients increase response and satisfaction with better counseling (Burban et al. 2013; Becker and Lois 1980). Another study indicates an increase in levels of frequency of counseling; monitoring and direct counseling also had a greater satisfaction rate (Liu et al. 1999).

Importance of the community pharmacist role was highlighted in a study conducted on Saudi Adults showing that poly-pharmacy (use of multiple medications more than is clinically needed) could be reduced through effective pharmacist’s counseling (Salih et al. 2013).

The impact of pharmacy services on Saudi community as previous research reveals that 44.6 % of the participants view pharmacists as an integral member of the healthcare system even though 38 % reported pharmacists were not asking about health related problems to solve medication-related hazards and 19.7 % reported that pharmacists gave unclear medication instructions (Al-Arifi 2012).

### Aim of the study

Our study aims to assess the satisfaction level of Saudi adults with the pharmaceutical services provided at the community pharmacies. This in turn will help us draw conclusions on the areas in need for improvement. The results provided from data analysis will provide better understanding of community pharmacy services provided for the Saudi community. We expect more than 50 % of the participants would be satisfied with Saudi community pharmacy services.

### Ethical approval

Ethical approval was granted by the Ethics Committee at King Fahad Medical City, Riyadh.

## Methods

### Study design

This cross-sectional study is designed to assess the general public satisfaction with the services provided by community pharmacies in different areas of Riyadh, Saudi Arabia. Sample selected from each area was proportional to population density (http://www.riyadh.gov.sa/en/pages/riyadhcity.aspx).

After a pilot study on ten individuals to ensure questions were properly phrased, six hundred and fifty questionnaires were distributed to adults in community pharmacies and public places around Riyadh. A modified version that contained questions of sections about patients’ views and satisfaction towards community pharmacy was used to assess the outcomes of interest. Questions included, for instance, demographic data, whether pharmacist accurately explains prescription instructions and spends time counseling patient, whether pharmacist asks about medication and disease history prior to dispensing prescriptions and patients’ understanding of dosage regimen and confidence in inquiring the pharmacist.

### The questionnaire

The questionnaire was divided into three sections: Section 1 covered demographic information including age, gender, marital status, the level of education and residential area.

Section 2 contained a 3 point Likert scale (Satisfied, Neutral, and Unsatisfied) for overall assessment of satisfaction of participant towards community pharmacy services.

Section 3 aimed at evaluating the participants’ satisfaction towards the services provided by the pharmacist through detailed 12 binary questions (yes/no). A total information-seeking behavior was positively associated with twelve questions that had to be answered with either “yes” for getting the right counseling techniques and “no” for not getting anything at all. For additional perceptions and comments, participants were given the chance to express them at the end of the questionnaire as the qualitative part of the research.

### Data analysis

The completed questionnaires were entered into SPSS Statistics version 20.0 (IBM Corporation, Somers, NY), and then descriptive analysis was performed. Categorical data was calculated as frequencies and percentages.

## Results

650 Surveys were distributed, and a total of 500 participants completely filled the survey with a response rate of 77 %. Also, 63 % of the participants were approached while at the pharmacy. Almost two-thirds of them (64.2 %) were university graduates. Our sample included 333 (66.6 %) females and 167 (33.4 %) males (Table 1).

**Table 1:** Demographic data of the participants

Demographic data	(n = 500)	(%)
Gender		
Female	333	66.6
Male	167	33.4
Level of education		
School	83	16.6
University	321	64.2
Postgraduate	96	19.2
Area		
West	54	10.8
East	167	33.4
North	83	16.6
South	51	10.2
Center	145	9.0

41 % of participants reported being satisfied with overall community pharmacy services while 26 % were unsatisfied, and 33 % evaluated community pharmacy neutrally. Table 2 depicts overall participants’ satisfaction with community pharmacy services.

**Table 2:** Measure of overall participants’ satisfaction

Satisfaction with pharmacy services	Number	Percentage
Satisfied	206	41.21
Neutral	165	32.91
Unsatisfied	129	25.88

Almost 57 % of satisfied group reported pharmacist asking about their concomitant medication, in contrast to the majority of unsatisfied and neutral groups (91 and 77 %, respectively) who reported not receiving this service; the differences were significant (p < 0.001). 59 % of the satisfied group also were asked about their comorbid diseases by the community pharmacist while majority of unsatisfied and neutral groups did not receive this service (82 % and 63 % respectively); the differences were significant (p < 0.001). 66 % of the satisfied group reported their community pharmacist ensured their understanding of dosage regimen, in contrast to 85 % of the unsatisfied and 71 % of the neutral group respectively; the differences were significant (p < 0.001).

The majority of the satisfied and neutral group (73 % and 52 % respectively) reported receiving significantly more counseling time than the unsatisfied group (24 %); the difference was significant (p < 0.001). 73 % of the satisfied group and 54 % of the neutral group agreed that they were able to discuss all medication related fears with the community pharmacist; while 54 % of the unsatisfied group reported not receiving this service (p < 0.001).

97 % of satisfied group and almost 80 % of the neutral group agreed significantly that patient counseling they received at a community pharmacist is a useful service while 58 % of the unsatisfied group disagreed (p < 0.001). The majority of the satisfied and neutral group reported their community pharmacist is available for counseling service while 78 % of the unsatisfied group disagreed (p < 0.001).

The majority in all of the satisfied, neutral and unsatisfied groups significantly agreed that community pharmacists explain the dosage regimen (96, 85 and 70 % respectively with p < 0.001). However, the majority of the satisfied, neutral and unsatisfied groups agreed that the community pharmacist did not explain the side effect of the medication (52, 87 and 98 % respectively with p < 0.001). Table 3 demonstrates the detailed findings.

**Table 3:** Patient satisfaction of pharmacy services

Response	Neutral group	Satisfied group	Unsatisfied group	Total	p value
Pharmacist self introduction					
No	158 (96.1)	193 (93.8)	124 (96.3)	476 (95.2)	0.619
Yes	6 (3.9)	13 (6.2)	5 (3.7)	24 (4.8)	
Pharmacist asks on current medication					
No	126 (76.7)	89 (43.4)	118 (91.4)	334 (66.8)	<0.001
Yes	38 (23.3)	117 (56.6)	11 (8.6)	166 (33.2)	
Asks comorbid diseases					
No	104 (63.1)	85 (41.1)	105 (81.5)	294 (58.8)	<0.001
Yes	61 (36.9)	121 (58.9)	24 (18.5)	206 (41.2)	
Explains dosage regimen					
No	26 (15.5)	8 (3.9)	38 (29.6)	72 (14.4)	<0.001
Yes	139 (84.5)	198 (96.1)	91 (70.4)	428 (85.6)	
Explains side effects					
No	144 (87.4)	107 (51.9)	126 (97.5)	377 (75.4)	<0.001
Yes	21 (12.6)	99 (48.1)	3 (2.5)	123 (24.6)	
Discussion on medication fears					
No	75 (45.6)	56 (27.1)	70 (54.3)	201 (40.3)	<0.001
Yes	89 (54.4)	150 (72.9)	59 (45.7)	299 (59.7)	
Ensures understanding of dosage					
No	117 (70.9)	70 (34.1)	110 (85.2)	297 (59.4)	<.001
Yes	48 (29.1)	136 (65.9)	19 (14.8)	203 (40.6)	
Appropriate time spent counseling					
No	78 (47.6)	56 (27.1)	99 (76.5)	233 (46.6)	<0.001
Yes	86 (52.4)	150 (72.9)	30 (23.5)	267 (53.4)	
Successful understanding of medication usage					
No	56 (34.0)	27 (13.2)	80 (61.7)	163 (32.6)	<0.001
Yes	109 (66.0)	179 (86.8)	50 (38.3)	337 (67.4)	
Useful patient counseling					
No	56 (21.4)	10 (3.1)	120 (58.0)	186 (23.3)	<0.001
Yes	103 (78.6)	125 (96.9)	86 (42.0)	314 (76.7)	
Able to discuss health and medications.					
No	81 (49.5)	51 (24.8)	86 (66.7)	219 (43.8)	<0.001
Yes	83 (50.5)	155 (75.2)	43 (33.3)	281 (56.2)	
Availability of dedicated pharmacist for counseling					
No	61 (36.9)	53 (25.6)	101 (77.8)	214 (42.8)	<0.001
Yes	104 (63.1)	153 (74.4)	29 (22.2)	286 (57.2)	

45 Participants added additional comments at the end of the questionnaire. 17 Commented on generic drug dispensing by the pharmacists without participant consent; 2 reported unclear information on medication; 23 commented on business like attitude of private sector community pharmacists and 3 commented on questionnaire’s questions.

## Discussion

In this exploratory study, we assessed the perception of Saudi community towards the pharmacist’s role as a health care provider. The main findings of this study reveal that the extent of satisfaction of Saudi adults towards community pharmacy services seems to be mainly attributed to effective communication between the pharmacist and the customer regarding information on medication, comorbid diseases, and concomitant medications. The ability of the study participants to discuss their medication and health related issues with the pharmacist was associated with higher satisfaction levels. This may be linked to the culture of the Saudi Adults who seem to prefer an open discussion of medication fears and health status with the pharmacist.

A notable finding was that among all groups the majority of community pharmacists appropriately explained the dosage regimen but did not explain side effects. Further investigations should be conducted on the training that pharmacists receive in private community pharmacies and whether it is aligned with the Saudi Ministry of Health requirements for good pharmaceutical practice guidelines.

The availability of the pharmacist to provide patient counseling services was an important determinant of satisfaction (74 %) and dissatisfaction (78 %) of the participants. However, further research should be conducted to determine whether the number of workforce per pharmacy may significantly affect available time allocated for each pharmacy customer and thus the quality of service provided.

Concerning Saudi Arabia, pharmacy sector faces a shortage of qualified practitioners and academic personnel. For instance, the Saudi Arabian Manpower Council reports that at least 17,000 pharmacists are needed by 2026 (Kheir et al. 2008). Community pharmacists in KSA were viewed in a study as a source of 50 % of OTC medications, 39 % of prescriptions, 27 % of dietary supplements and 14 % of diagnostic services. Community pharmacy services in Saudi Arabia vary according to the location. For instance, major cities such as Riyadh and Jeddah had pharmacies offering dispensing of prescription medicines, a vast array of OTC drugs, cosmeceutical products and branded cosmetics sales in addition to diagnostics services such as weight, height, BMI, blood pressure and blood sugar levels. On the other hand, some of these services are not present in rural cities of Saudi Arabia where the pharmacies tend to provide only essential drugs for prescription dispensing with minimal OTC and cosmeceuticals (Al-Hassan 2009).

The notable negative perceptions among participants on pharmacist–patient counseling services may be attributed to potential factors: lack of enforcement of national regulations, suboptimal compliance to the code of ethics and professionalism among community pharmacists as well as financial interests of community pharmacists as they tend to be owned by the private sector (Bawazir 1992; Anderson et al. 2004).

Among the participants’ comments at the end of the questionnaire was the issue of generic drugs, where the pharmacist picks a generic brand and dispenses the medication without discussing what other options are available; such as the availability of the original brand medication or lower cost generic brand. This presents a need for professional training for community pharmacists to take patient/pharmacy customer opinion into consideration if he demanded the brand drug or a generic of lower/higher price.

Moreover, some complained that the pharmacists were business-oriented instead of being oriented towards patient care. This presents the need for government regulated training for community pharmacists in the private sector and enforced follow up on private sector community pharmacies to ensure that the quality of patient counseling provided is optimal and hence minimizes potential exposure to risks of dosage mistakes and drug–drug interactions.

Other concerns on community pharmacy services were highlighted in a study from the Eastern Province of Saudi Arabia. It demonstrated a high rate of antibiotic sales without a prescription for presumed mild cases of infections which show a lack of adherence to MOH regulations. This presented hazards to the Saudi community as it was reported that none of the visited pharmacists asked about history of drug allergy or provided information regarding the potential drug interactions when dispensing any antibiotics for any of the simulated clinical scenarios (Abdulhak et al. 2011).

Our results are in line with similar research conducted in developing countries. In a study carried out in Pakistan, it was found that 84.1 % participants agreed that pharmacists are important to offer patient centered services; the pharmacists need to understand fully patients’ perspectives and views to meet their needs and this was the major influencing factor of pharmacy services (Khan et al. 2013).

This shows that developing countries, regardless of their economic status; have many obstacles to overcome to deliver effective pharmacist–patient counseling to their communities. The contrast between developed and developing countries pharmacies as studies indicate seems to depend on awareness and community culture.

These results are in line with western population perception on the importance of community pharmacist’s role. For instance; a Norwegian study showed that adults are at a greater risk for general harm of medicines. Their concern was about getting the right counseling and proper use of their medication to decrease harm to the patients (Viktil et al. 2013).

Our results recommend that to increase patient satisfaction, we should train pharmacists to improve their counseling techniques, which in turn will lead to better professional performance of community pharmacies thus improving the Saudi Adults’ quality of life.

This could be achieved by government-initiated training where community pharmacists take an active role to consider development of patient counseling as an indispensable service provided to the Saudi community. Likewise, National administration of Pharmacists Syndicate should collaborate with privately owned pharmacies as they represent a significant portion of community pharmacists to ensure medications are dispensed with complete information about dosage, contraindications, and drug–drug interactions.

The limitations of the study were the small sample size and bias of the participants in answering the questionnaire. Further studies need to be carried out in Saudi Arabia with a greater sample size while taking into account the results of patient satisfaction after increased pharmacist counseling, especially in the regions where satisfaction level was less. This would help to gain a better perception and lead to an improvement of the community pharmacy services of Saudi Arabia.

## Conclusion

Saudi adults show different perceptions towards community pharmacy services. Nevertheless, the overall level of satisfaction showed that most Saudi patients are satisfied with the services provided, some negative perceptions on the quality of patient counseling highlights the need for effective pharmacists’ patient counseling training programs.
